# CircVPRBP inhibits nodal metastasis of cervical cancer by impeding RACK1 O-GlcNAcylation and stability

**DOI:** 10.1038/s41388-023-02595-9

**Published:** 2023-01-19

**Authors:** Chunyu Zhang, Hongye Jiang, Li Yuan, Yuandong Liao, Pan Liu, Qiqiao Du, Chaoyun Pan, Tianyu Liu, Jie Li, Yili Chen, Jiaming Huang, Yanchun Liang, Meng Xia, Manman Xu, Shuhang Qin, Qiaojian Zou, Yunyun Liu, Hua Huang, Yuwen Pan, Jiaying Li, Junxiu Liu, Wei Wang, Shuzhong Yao

**Affiliations:** 1 Department of Obstetrics and Gynecology, the First Affiliated Hospital, Sun Yat-sen University, 510080 Guangzhou, Guangdong China; 2 Department of Biochemistry and Molecular Biology, Zhongshan School of Medicine, Sun Yat-sen University, Guangzhou, 510080 China

**Keywords:** Cervical cancer, Lymphangiogenesis, Metastasis

## Abstract

Lymph node (LN) metastasis is one of the most malignant clinical features in patients with cervical cancer (CCa). Understanding the mechanism of lymph node metastasis will provide treatment strategies for patients with CCa. Circular RNAs (circRNA) play a critical role in the development of human cancers. However, the role and mechanism of circRNAs in lymph node metastasis remain largely unknown. Here, it is reported that loss expression of circRNA circVPRBP was closely associated with LN metastasis and poor survival of CCa patients. In vitro and in vivo assays showed that circVPRBP overexpression notably inhibited lymphangiogenesis and LN metastasis, whereas RfxCas13d mediated silencing of circVPRBP promoted lymphangiogenesis and the ability of the cervical cancer cells to metastasize to the LNs. Mechanistically, circVPRBP could bind to RACK1 and shield the S122 O-GlcNAcylation site to promote RACK1 degradation, resulting in inhibition of Galectin-1 mediated lymphangiogenesis and LN metastasis in CCa. Taken together, the results demonstrate that circVPRBP is a potential prognostic biomarker and a novel therapeutic target for LN metastasis in CCa patients.

## Introduction

Cervical cancer (CCa) is the fourth most common female malignancy worldwide [1]. One of the main hitches of low therapeutic efficacy is that CCa patients often develop lymph node (LN) metastasis even in the early stage [2, 3]. CCa patients with multi-nodal metastasis show particularly low benefits from surgery and radiotherapy, and the 5-year-survival rate of CCa patients could decrease from 95% to 33.3% once upon lymphatic spread occurance [3]. Pelvic LN metastasis as a critical independent prognostic factor, is one of the leading causes of cervical cancer death [4]. With mounting evidence that the lymph nodes are foothold for further tumor dissemination [5, 6], elucidating the mechanisms underlying LN metastasis in CCa is of paramount importance.

LN metastasis is a multi-process and complicated biology event containing both lymphangiogenesis in primary tumor and invasiveness of tumor cells, which finally favor entry of tumor cells into the lymphatic vasculature [7]. Lymphangiogenesis is an important initial step and essential event during cancer cells lymphatic metastasis [8, 9]. Cervical cancer cells could enhance the remodeling of lymphatic vessels by secreting pro-lymphangiogenic growth factors, such as the VEGF-C [10] and Galectin-1 [11]. In the meantime, cervical cancer cells somehow acquire the driving force to erode the extracellular matrix and the motility to extravasate into the new developed lymphatic vessels [12]. However, it is not currently clear how to effectively prevent the formation of lymphangiogenesis and inhibit the invasiveness of cervical cancer cells.

O-linked β-N-acetylglucosamine (O-GlcNAc) is a dynamic post-translational modification on serine or threonine residues of proteins, catalyzed by O-GlcNAc transferase (OGT) [13]. O-linked β-N-acetylglucosaminylation (O-GlcNAcylation) regulates the activities of a wide range of proteins involved in cancer-relevant processes [14–16]. Previous studies indicates that aberrant protein O-GlcNAcylation modification might be associated with LNM progression in invasive ductal breast carcinoma, and O-GlcNAcylation could promote colorectal cancer metastasis by enhancing EZH2 protein stability and function [17, 18]. Nevertheless, little is currently known about the specific mechanisms of O-GlcNAcylation modification in LN metastasis of cancer cells. The receptor for activated C-kinase 1 (RACK1) is a member of the Trp-Asp repeat protein family, and it has been widely accepted as a multifaceted scaffolding protein involved in different biological events in cancer progression, such as cell migration and angiogenesis via interaction with different partners [19–21]. It has been reported that RACK1 could participate in the lymphangiogenesis and LN metastasis of cervical cancer in a Galectin-1-dependent manner [11], suggesting that targeting RACK1 may be a promising strategy. Meanwhile, biochemical studies have established that O-GlcNAcylation modifications of RACK1 can regulate its stability, thus promoting hepatohepatocellular carcinogenesis [22]. Unveiling the molecular mechanism underlying RACK1 expression or its O-GlcNAcylation will hopefully open new avenues to prevent or overcome nodal metastasis of cervical cancer. However, it is unclear how this regulation occurs in cervical cancer.

Circular RNAs (circRNAs) are generated from back-splicing of pre-mRNAs to form covalently closed transcripts [23, 24]. CircRNA expression is not only conserved very well among species, but also highly cell-type and tissue specific [25]. In addition, circRNAs are more stable than related linear mRNAs. Accumulating evidence indicates that circRNAs may involve in the progression of many cancers, including breast cancer [26], glioblastoma [27], cervical cancer [28], et.al. We have previously reported that circular RNA hsa_circ_0043280 could inhibit cervical cancer LN metastasis through miR-203a-3p/PAQR3 axis [29]. Circular RNAs could function as miRNAs sponge or protein scaffold which led to the regulation of genes expression involved in CCa progression [30–32]. Of note, the specificity of RACK1 interaction with circRNAs is expected for at least some of its regulation and biological function in cancer cells, which require further studies. Moreover, the biological functions and clinical significance of circRNAs in nodal metastasis of CCa remain largely unknown, warranting further exploration.

In this work, we uncover a new regulatory mechanism of nodal metastasis in CCa. We report a novel circRNA, circVPRBP, which was significantly downregulated in nodal metastatic CCa tissues and cell lines. Low expression of circVPRBP was closely associated with tumor size, LN metastasis, lymphovascular space invasion (LVSI) and poor survival of CCa patients. Subsequently, we demonstrated that overexpression circVPRBP markedly inhibited the nodal metastasis and lymphangiogenesis of CCa in vivo and in vitro. Mechanistically, circVPRBP could bind to RACK1 and shield a S122 O-GlcNAcylation site to promote RACK1 degradation. Therefore, our finding revealed that circVPRBP might be a potential prognostic biomarker and a novel therapeutic target for LN metastasis in CCa patients.

## Results

### CircVPRBP expression is downregulated during CCa LN metastasis

**Fig. 1:**
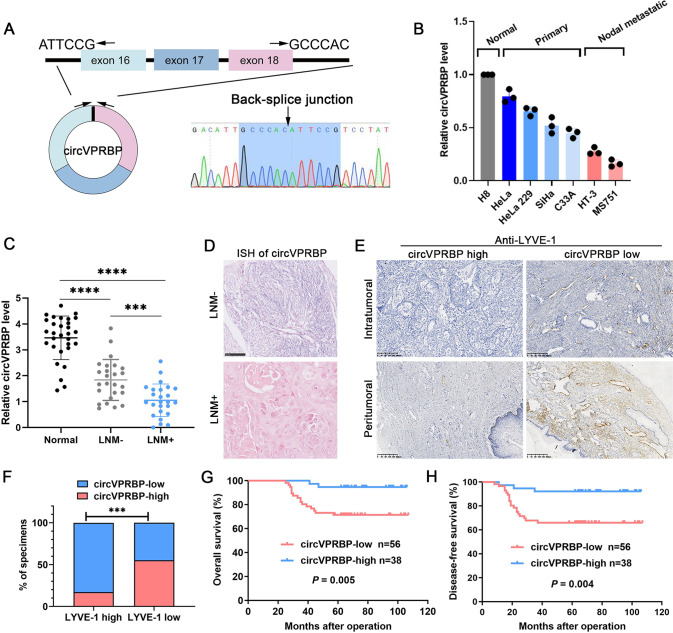
CircVPRBP expression is downregulated during CCa LN metastasis. **A** The genomic locus of circVPRBP. The back splicing junction was identified by Sanger sequencing. **B** Relative expression of circVPRBP in CCa cell lines and a normal cervix cell line H8. **C** RT-qPCR analysis of circVPRBP expression in normal cervix tissues and CCa samples. **D** ISH analysis of circVPRBP in CCa primary tumors with or without LN metastasis. Original magnifification, ×400. **E** Representative images of tissue samples with lymphatic vessels in intratumoral (upper panels) and peritumoral (lower panels) areas of CCa with low or high expression of circVPRBP. The expression levels of circVPRBP were quantified by ISH, and lymphatic vessel density was quantified by immunohistochemistry using the anti-LYVE-1 antibody. Original magnifification, ×100. **F** The percentages of specimens with high or low levels of LYVE-1-positive lymphatic vessels in CCa with low or high expression of circVPRBP. **G**, **H** Kaplan–Meier analysis showed the negative correlation between circVPRBP expression levels and the overall survival (G) and disease-free survival (H) in our cohort. Each experiment was performed at least three times independently. ***P < 0.001; ****P < 0.0001.

Our previous study has identified circVPRBP (hsa_circ_0065898) as a 367-base circRNA down-regulated in CCa [29], as sanger sequence showed the back splicing site of the circVPRBP (Fig. 1A). To explore the function of circVPRBP in CCa, we began by examining its expression in CCa cell lines by quantitative real-time polymerase chain reaction (RT-qPCR) with divergent primers. We found that circVPRBP had a decreased expression in CCa cell lines relative to normal cervix cell line H8. Moreover, circVPRBP was more abundant in primary tumor derived cell lines HeLa, HeLa229, SiHa, C33A cells than metastatic lymph node derived cell lines HT-3 and MS751 (Fig. 1B). Consistent with this trend, in a cohort of fresh-frozen CCa patient samples, circVPRBP level was significantly lower in the tumor samples than in non-tumor tissues. Moreover, we observed progressive loss of circVPRBP expression in lymph node metastatic samples (Fig. 1C).

Then we expanded our survey in another cohort of CCa samples (*n* = 94) through RNA in situ hybridization on primary tumor slices. Intriguingly, we observed that loss of circVPRBP expression was more common in primary tumors with LN metastasis (Fig. 1D). Intriguingly, LYVE-1 staining showed that the number of lymphatic vessels was robustly increased in intratumoral and peritumoral areas of CCa with low circVPRBP expression (Fig. 1E, F), suggesting that circVPRBP may play a vital role in lymphangiogenesis and LN metastasis in cervical cancer.

Within the primary CCa samples, circVPRBP loss was associated with prognostic clinical factors, including tumor size (*P* = 0.023), lymphovascular space invasion (*P* = 0.020), and LN metastasis (*P* = 0.014), but not with age at diagnosis, pathologic types, differentiation, et al. (Supplementary Table S1). Furthermore, Kaplan–Meier survival curves and log-rank test analyses showed that low abundance of circVPRBP in these samples associated with shorter overall survival (OS) and disease free survival (DFS) (Fig. 1G, H). In addition, univariate and multivariate analysis suggested that circVPRBP expression (95% CI: 0.040–0.825; *P* = 0.027), tumor size (95% CI: 1.798–13.293; *P* = 0.002), lymphovascular space invasion (95% CI: 1.271–9.398; *P* = 0.015) and LN metastasis (95% CI: 1.327–9.285; *P* = 0.011) were independent prognostic for OS of cervical cancer patients. Similarly, circVPRBP expression (95% CI: 0.068–0.829; *P* = 0.024), tumor size (95% CI: 1.752–10.777; *P* = 0.002), lymphovascular space invasion (95% CI: 1.559–9.890; *P* = 0.004) and LN metastasis (95% CI: 1.305–7.369; *P* = 0.010) were independent prognostic for DFS of cervical cancer patients **(**Supplementary Tables S2, 3). Collectively, our data show that circVPRBP expression is reduced in CCa, and its loss associates with LN metastasis and patient outcomes. These results suggest that circVPRBP loss may contribute to CCa LN metastasis, and its expression may have prognostic value for CCa patients.

### Characterization of circVPRBP in CCa cells

We designed divergent primers for circVPRBP and convergent primers for VPRBP and circular form of circVPRBP could only be amplified in cDNA but not in gDNA (Fig. S1A). Meanwhile, we found that circVPRBP expression level was more stable than VPRBP mRNA after Actinomycin D treatment at indicated several time points (Fig. S1B). Since circular RNAs don’t have 3’ poly adenylated tail, we used oligo dT primers or random primers to make reverse transcript products from SiHa and MS751 cells and then detect circVPRBP expression, and circVPRBP was undetectable in oligo dT reverse transcribed cDNA but existed in random primers treated products (Fig. S1C), and circVPRBP could still be detected after RNase R treatment which could digest linear RNA (Fig. S1D).

### CircVPRBP inhibits invasion of CCa cells in vitro and LN metastasis of CCa in vivo

**Fig. 2:**
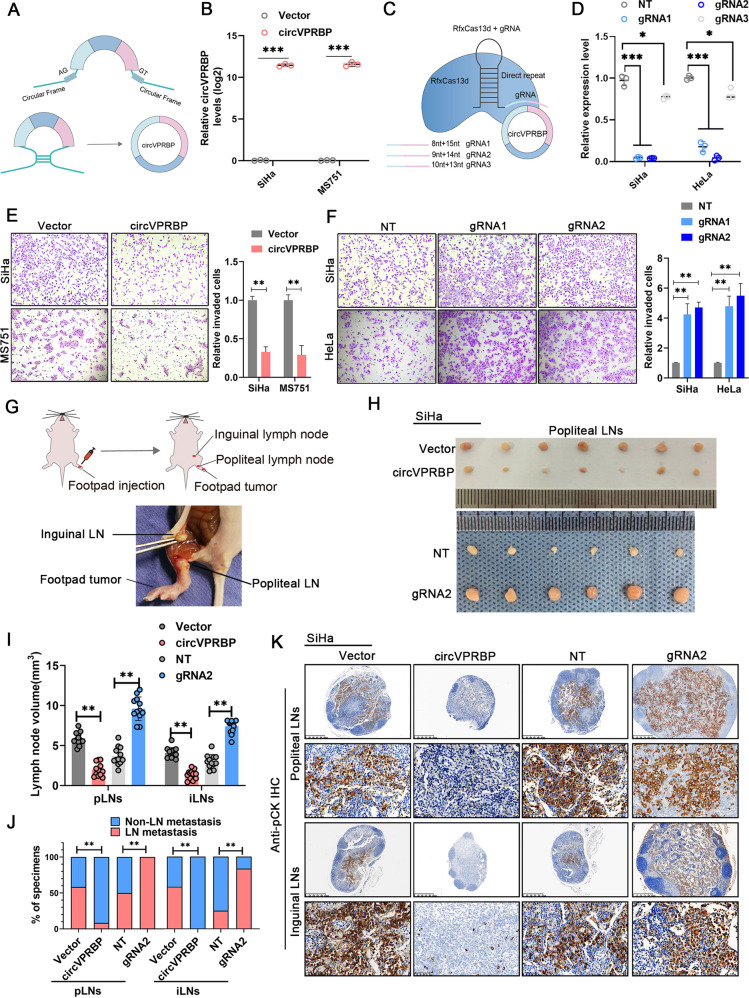
CircVPRBP inhibits invasion of CCa cells in vitro and LN metastasis of CCa in vivo. **A**, **B** The schematic illustration of circVPRBP expression vector, and the expression levels of circVPRBP in SiHa and MS751 cells stably transfected with circVPRBP or corresponding negative control were detected by RT-qPCR. **C**, **D** Schematic of circRNA knockdown using RfxCas13d–BSJ-gRNA system. Three BSJ-gRNAs targeting the BSJ site were designed for circVPRBP. The knockdown efficiency for each gRNA of circVPRBP was evaluated by RT-qPCR. BSJ, back splicing junction. **E**, **F** Invasion assays for SiHa and MS751 cells with circVPRBP overexpression or knockdown. Original magnifification, ×100. **G** The schematic illustration of in vivo nude mouse LN metastasis model of CCa. **H** Representative images of popliteal LNs and inguinal LNs of indicated groups (*n* = 12). **I** Histogram analysis showed the LNs volume in different groups. **J** Quantification of the rate of LN metastasis in the indicated groups. **K** Representative images of immunostaining of pan-cytokeratin of popliteal LNs and inguinal LNs. Original magnifification, ×100. *P < 0.05; **P < 0.01; ***P < 0.001.

According to circVPRBP expression in cervical cancer cell lines (Fig. 1B), the SiHa and MS751 cell line were selected for overexpression of circVPRBP (Fig. 2A, B). To specifically and effectively silence circVPRBP, we used RfxCas13-gRNA mediated circRNA knockdown system and successfully silenced circVPRBP expression in SiHa and HeLa cells (Fig. 2C, D). Transwell assay showed that circVPRBP overexpression could abolish the invasiveness of CCa cells and knockdown circVPRBP effectively promoted the invasion ability (Fig. 2E, F), suggesting circVPRBP could repress tumor metastasis.

To investigate the impact of circVPRBP on LN metastasis of cervical cancer, an in vivo nude mouse LN metastasis model was employed (Fig. 2G), which simulates the directional drainage and metastasis of lymph nodes of cervical cancer. The CCa cells were implanted into the footpads of nude mice, the popliteal and inguinal lymph nodes were removed and analyzed. Strikingly, circVPRBP overexpression notably inhibited LN metastasis. Conversely, silencing circVPRBP promoted the ability of the cervical cancer cells to metastasize to the LNs (Figs. 2H, S2A, B). The volumes of the popliteal and inguinal LNs were smaller in the circVPRBP tumor group than in the control group, whereas the volumes of the LNs were significantly larger in the gRNA-circVPRBP group than in the control group (Figs. 2l,S2C). Immunostaining of pan-cytokeratin confirmed that forced expression of circVPRBP significantly repressed the lymphatic metastatic ability of cervical cancer cells and ablation of circVPRBP augmented LN metastasis (Figs. 2J, K, S2D, E). Together, these findings suggest that circVPRBP could inhibit LN metastasis of cervical cancer in vivo.

### CircVPRBP represses lymphangiogenesis in vivo and in vitro

**Fig. 3:**
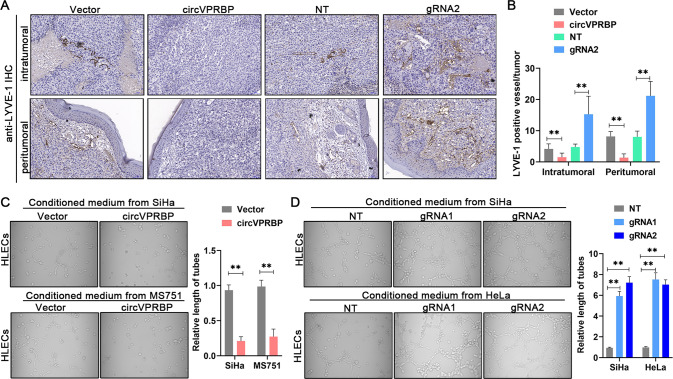
CircVPRBP represses lymphangiogenesis in vitro and in vivo. **A**, **B** Representative images of intratumoral and peritumoral lymphatic vessels stained with anti-LYVE-1 (A) and histogram quantification of LYVE-1 positive lymphatic vessels (B) in the indicated groups. Original magnifification, ×100. **C**, **D** Representative images (left panels) and quantifications (right panels) of tube formation by HLECs treated with conditioned medium collected from CCa cells with circVPRBP overexpression or knockdown. Each experiment was performed at least three times independently. **P < 0.01.

Lymphangiogenesis is functionally important in LN metastasis which has led to the idea that blocking them, by targeting lymphangiogenic signaling pathways, might be a useful therapeutic strategy to restrict metastatic spread [33]. Since circVPRBP was negatively correlate with LVSI and lymphatic vessel density in primary tumors, we employed experiments to explore whether circVPRBP influenced lymphangiogenesis. To test the possibility, immunohistochemistry (IHC) analyses using an antibody to a lymphatic marker, LYVE-1, were performed to quantify of intratumoral and peritumoral lymphatic vessels in the primary tumors. It turned out that the LYVE-1 positive vessels significantly declined in the mice bearing circVPRBP overexpression cells and increased in the mice inoculated with circVPRBP ablation cells (Fig. 3A, B), indicating that circVPRBP inhibited lymphangiogenesis in vivo. To further elaborate the functional impact of circVPRBP on lymphangiogenesis in vitro, we pursued tube formation assay. Expectedly, conditioned medium derived from circVPRBP over-expressed SiHa and MS751 cells obviously restrained tube formation by human lymphatic endothelial cells (HLECs), whereas loss of circVPRBP remarkably increased the HLECs tube formation capability (Fig. 3C, D).

**Fig. 4:**
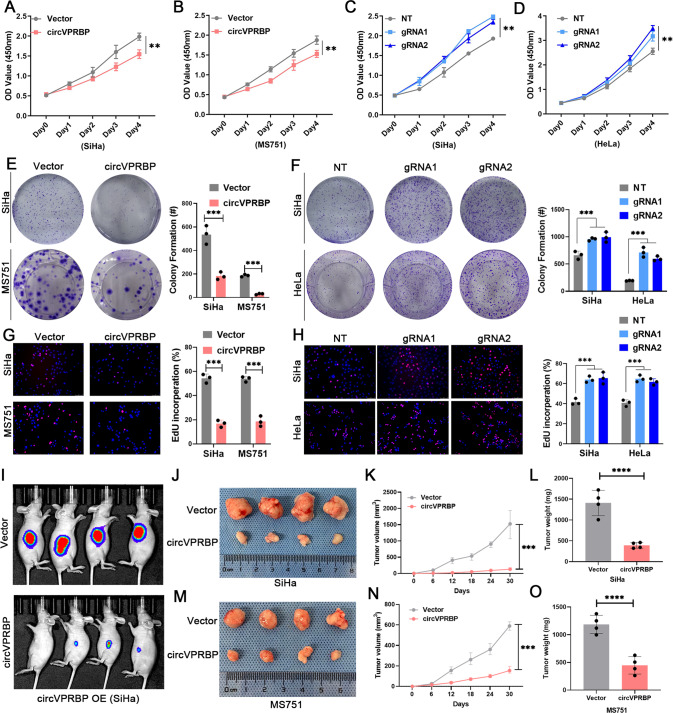
circVPRBP suppresses CCa tumorigenesis in vitro and in vivo. **A–D** The proliferative abilities of SiHa and MS751 cells were measured by the CCK-8 assay after overexpression of circVPRBP and knockdown of circVPRBP. **E**–**H** Colony formation and EdU assays for CCa cells with circVPRBP overexpression or knockdown. Original magnifification, ×100. **I**–**O** Representative images and tumor growth curves and tumor weight of nude mouse of subcutaneous tumors in different treatment groups. Each experiment was performed at least three times independently. **P < 0.01; ***P < 0.001; ****P < 0.0001.

Tumorigenicity is a major factor correlated with lymphangiogenesis and LN metastasis in various solid tumors. Therefore, we investigated the tumorigenic effect of circVPRBP in CCa. Cell Counting Kit-8 (CCK-8), colony formation assays and EdU proliferation assays revealed that circVPRBP overexpression decreased proliferation and colony formation in CCa cells. Conversely, circVPRBP knockdown yielded the opposite effect on proliferation and colony formation (Fig. 4A–H). Then, we constructed a subcutaneous xenograft model to evaluate the tumorigenic capacity of circVPRBP in vivo. Our results indicated that circVPRBP overexpression decreased the tumor growth of CCa (Fig. 4I). Moreover, tumors in the circVPRBP overexpression group were of lower weight and size than those in the control group (Fig. 4J–O). Taken together, these results support the fact that circVPRBP could inhibit lymphangiogenesis in cervical cancer.

### CircVPRBP interacts with RACK1 to inhibit RACK1 protein expression

**Fig. 5:**
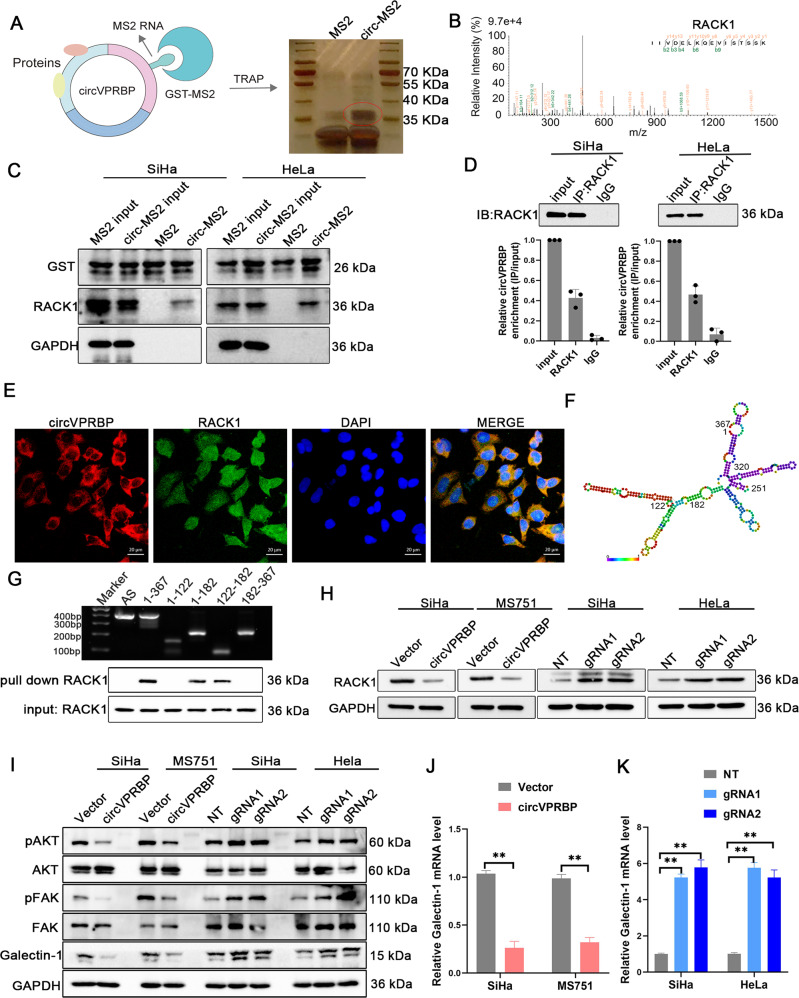
CircVPRBP interacts with RACK1 to inhibit RACK1 protein expression. **A** Diagram for TRAP assay. **B** Mass spectrometry identified RACK1 was pulled down from HeLa cells lysates by MS2-circVPRBP. **C** Western blots of RACK1 pulled down by circVPRBP in TRAP assay. **D** Binding of circVPRBP to RACK1 in SiHa and HeLa cells was detected by RIP assay. **E** Fluorescence colocalization assay showed that circVPRBP and RACK1 protein colocalize in the cytoplasm of SiHa cells. **F** The predicted secondary structure of circVPRBP using the RNAfold WebServer, based on the minimum free energy. Color scales indicated the confidence of predictions for each base, and the red shades demonstrated the predictions with strong confidence. **G** RNA pulldown assay showed RACK1 pulled down by biotin-labeled circVPRBP of different lengths. **H** Protein levels of RACK1 in indicated CCa cells. **I** Western blot analysis of the protein levels of pAKT, AKT, pFAK, FAK, Galectin-1 in the indicated cells. **J**, **K** Galectin-1 mRNA expression levels in circVPRBP overexpression or knockdown cells. Each experiment was performed at least three times independently. ns, no significant; **P < 0.01.

To observe cellular localization of the circVPRBP, we conducted qRT-PCR analysis for nuclear and cytoplasmic circVPRBP. Results showed that circVPRBP mainly located in the cytoplasm (Fig. S3A, B). To gain mechanistic insights, we performed Tagged RNA affinity purification (TRAP) assay using MS2-labeled circVPRBP as bait to identify circVPRBP-interacting proteins in HeLa cells, an evident band with a molecular weight between 35 and 40 kDa was subjected to mass spectrometry (MS), which highlighted RACK1, a member of the Trp-Asp repeat protein family (Figs. 5A, B). We confirmed circVPRBP could interact with endogenous RACK1 in CCa cells through TRAP-western blotting and RNA immunoprecipitation (RIP) assays (Fig. 5C, D). Besides, fluorescence colocalization results showed that circVPRBP and RACK1 protein colocalize in the cytoplasm of SiHa and HeLa cells (Figs. 5E, S3C). Moreover, RNA pull-down and using various truncated constructs of circVPRBP molecules revealed that the RACK1 proteins intensively interacted with segment 122–182 nt of circVPRBP (Fig. 5F, G). Indeed, overexpressing circVPRBP hardly affected the mRNA expression of RACK1, whereas markedly decreased RACK1 protein levels (Figs. S3D, 5H). By contrast, silencing circVPRBP significantly provoked the protein level rather than the mRNA level of RACK1 (Figs. S3E, 5H). Previous literature verified that RACK1 could promote cervical cancer lymphangiogenesis and lymph node metastasis by augmenting Galectin-1-induced downstream FAK, and AKT signaling in cervical cancer cells [11]. We also found that overexpressing circVPRBP could suppress pAKT and pFAK expression, and abolished Galectin-1 production of CCa cells in both protein and mRNA levels, which could explain circVPRBP induced lymphangiogenesis inhibition, whereas knockdown of circVPRBP had the opposite trend (Figs. 5I–K). Together, these results show that circVPRBP could bind RACK1 and downregulate RACK1 protein expression.

### CircVPRBP destabilizes RACK1 by impeding a S122 O-GlcNAcylation site

**Fig. 6:**
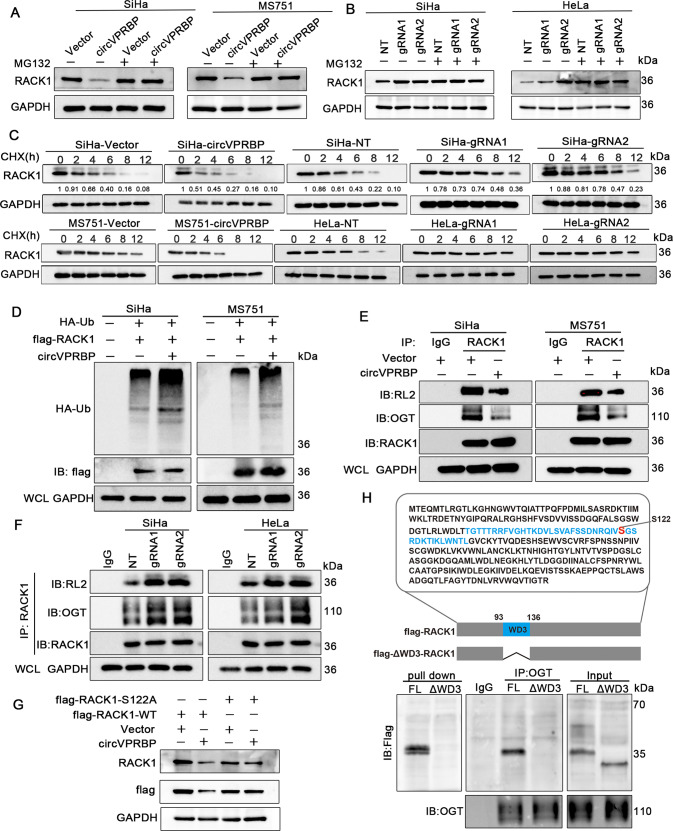
CircVPRBP destabilizes RACK1 by impeding a S122 O-GlcNAcylation site. **A**, **B** Western blots showed the expression levels of RACK1 in circVPRBP overexpression or knockdown CCa cells with or without MG132 treatment. **C** RACK1 expression levels in circVPRBP overexpression or knockdown CCa cells with or without CHX treatment at indicated time points. **D** The effect of overexpressing circVPRBP on the levels of polyubiquitination of RACK1 in SiHa or MS751 cells. **E**, **F** Interaction between RACK1 and OGT/O-GlcNAcylation(RL2) modification was evaluated by co-IP assays in indicated CCa cells. **G** Western blot analysis of RACK1 levels in 293 T cells with the indicated treatments. **H** Amino acid sequence of the RACK1. Location of WD3 region was highlighted in blue, and S122 O-GlcNAcylation sites are highlighted in red. Interaction between full length RACK1/ΔWD3-RACK1 and circVPRBP or OGT was evaluated by RNA pull down or co-IP assays in indicated CCa cells. Each experiment was performed at least three times independently.

To explore whether circVPRBP induced RACK1 downregulation through proteasome mediated protein degradation, we then used proteasome inhibitor MG132 to treat CCa cells before circVPRBP overexpression or ablation. Overexpressing or silencing circVPRBP hardly changed RACK1 protein levels when proteasome-mediated protein degradation was blocked by MG132 treatment (Fig. 6A, B). Moreover, overexpressing circVPRBP accelerated the degradation of RACK1 proteins, while silencing circVPRBP significantly extended protein half-life of RACK1 (Fig. 6C). Furthermore, overexpression of circVPRBP caused remarkable increase of polyubiquitination of RACK1 proteins in SiHa and MS751 cells (Fig. 6D), supporting the fact that circVPRBP could bind and destablize RACK1 in CCa cells.

Previous literatures have revealed that O-GlcNAcylation modification can influence the stability of RACK1 [22], so we wondered whether circVPRBP could affect O-GlcNAcylation of RACK1. Strinkingly, immunoprecipitation analysis was performed and revealed that overexpression of circVPRBP dramatically reduced the O-GlcNAcylation (RL2) modification level of RACK1, and reduced binding between O-linked N-acetylglucosamine transferase (OGT) and RACK1 (Fig. 6E). Meanwhile, silencing of circVPRBP increased RACK1 O-GlcNAcylation modification and conjunction with OGT (Fig. 6F). Moreover, we mutated the S122 O-GlcNAcylation sites of RACK1 and then co-transfected the S122A mutant variants together with Vector or circVPRBP plasmids in 293 T cells. Reconciling with our present findings, S122A mutation abrogated the circVPRBP-promoted RACK1 degradation, while there was significantly RACK1 degradation difference in the context of wild type RACK1 transfection (Fig. 6G). In order to demonstrate the relationship between circVPRBP-binding sites and O-linked N-acetylglucosamine transferase (OGT)-binding sites in RACK1, we constructed a flag-tagged RACK1 mutant without WD3 region (ΔWD3-RACK1-flag) since OGT mediated S122 site O-GlcNAcylation locates in the WD3 region of RACK1. RNA pull down assay showed that ΔWD3-RACK1 could not be pulled down by circVPRBP, whereas full length of RACK1 obtained the binding function with circVPRBP, suggesting WD3 region is circVPRBP-binding sites in RACK1. In the meantime, we performed IP experiment using anti-OGT antibody in CCa cells and the result showed that ΔWD3-RACK1 could not bind with OGT, which means that WD3 region contains OGT-binding sites in RACK1 (Fig. 6H). The results above indicates that WD3 region of RACK1 is the common binding sites with both circVPRBP and OGT, which explains why overexpression of circVPRBP dramatically reduce the O-GlcNAcylation (RL2) modification level of RACK1, and reduce binding between OGT and RACK1. Taken together, these results demonstrate that circVPRBP could mediate RACK1 degradation via blocking S122 O-GlcNAcylation site of RACK1.

### CircVPRBP represses nodal metastasis of CCa in a RACK1-dependant manner

**Fig. 7:**
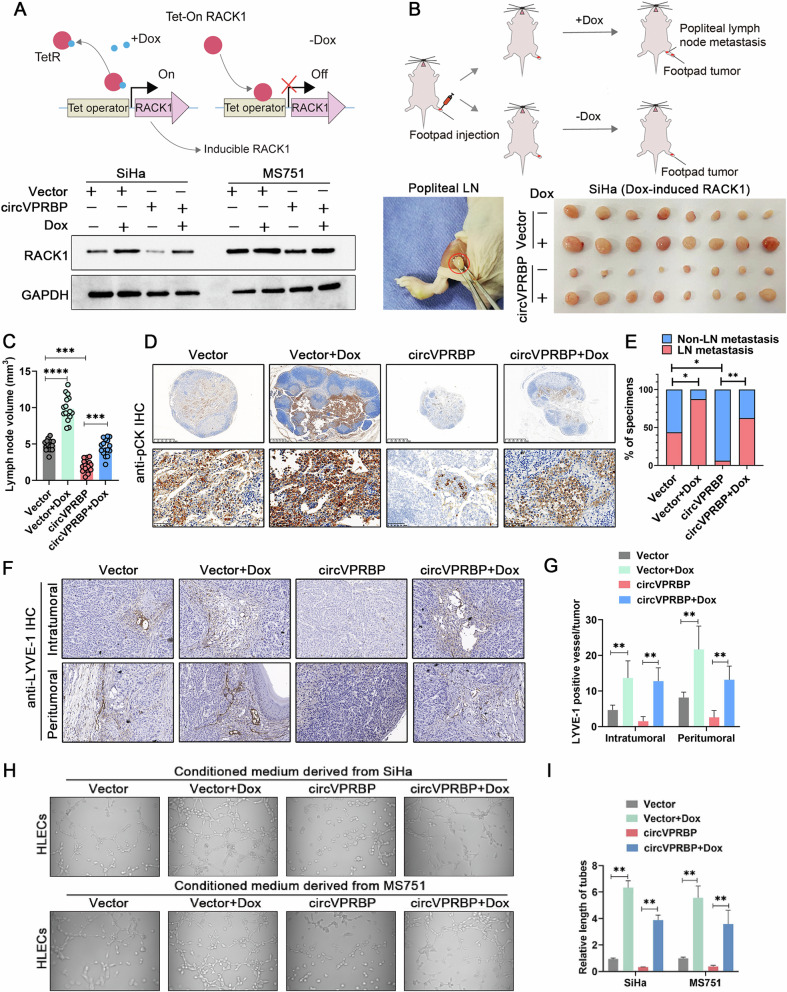
CircVPRBP represses nodal metastasis of CCa in a RACK1-dependant manner. **A** The schematic illustration of Tet-on induced RACK1 expression system (upper panel). Western blots analysis showed the RACK1 expression levels under indicated treatments (lower panel). **B** The workflow of the xenograft popliteal node metastasis model (upper panel). Representative images of popliteal LNs in different treatment groups (lower panel, *n* = 16). **C** The LNs volume were shown as the histogram analysis in different groups. **D** Representative images of immunostaining of pan-cytokeratin of popliteal LNs. Original magnifification, ×100. **E** Quantification of the rate of LN metastasis in the indicated groups. **F**, **G** LYVE-1 positive vessels in the footpad tumors of indicated groups as indicated by immunohistochemistry (F) and histogram analysis (G). **H**, **I** Tube formation by HLECs treated with conditioned medium collected from SiHa and MS751 cells under different treatments. Each experiment was performed at least three times independently. *P < 0.05; **P < 0.01; ***P < 0.001; ****P < 0.0001.

Next, we performed a range of rescue assays to investigate whether circVPRBP suppressed the lymphatic metastasis of CCa cells in a RACK1-dependent manner. Using a Tet-on inducible system in CCa cells, addition of doxycycline (Dox) could induce RACK1 expression in Vector or circVPRBP overexpression cells (Fig. 7A). We performed another set of xenograft popliteal node metastasis model to further investigate the role of circVPRBP and RACK1 in nodal metastasis of CCa. The results provide direct evidence that Dox-induced RACK1 reversed circVPRBP mediated nodal metastatic suppression, thus reconciling with the concept that blocking RACK1 degradation could be pivotal in CCa lymphatic metastasis (Fig. 7B).

We observed that the volume of the popliteal lymph nodes was significantly smaller in the circVPRBP overexpression group than that in the control group, and that the Dox-induced RACK1 groups showed a greater nodal volume than that in the both Vector and circVPRBP overexpression group (Fig. 7C). The immunostaining of cytokeratin confirmed that circVPRBP overexpression led to a significant inhibition in the metastatic capability of CCa cells to the popliteal lymph nodes and that the Dox-induced RACK1 rescued this trend, as determined by quantifying the number of metastatic lymph nodes (Fig. 7D, E). Furthermore, Dox-induced RACK1 greatly reversed the repressing effect of circVPRBP on lymphangiogenesis in vivo and in vitro (Fig. 7F–I). Taken together, these results support the fact that circVPRBP do inhibit CCa nodal metastasis and lymphangiogenesis through participating in RACK1 degradation.

**Fig. 8:**
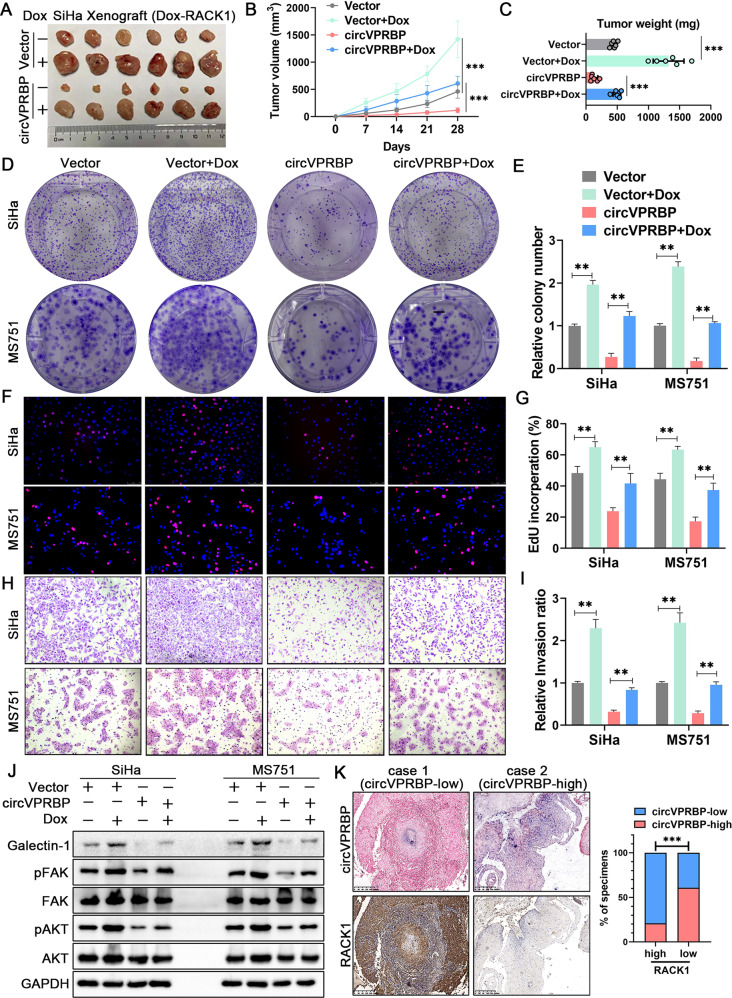
Blocking RACK1 degradation is crucial for tumor growth in CCa. **A–C** Representative images, tumor growth curves and tumor weight of subcutaneous tumors in indicated groups. **D–G** colony formation and EdU assays are performed to evaluate the proliferation ability of indicated cells. Original magnifification, ×100. **H, I** Transwell assay indicated the altered invasion ability under different treatments (left panel) and quantification by the histogram (right panel). **J** Western blot analysis of the protein levels of Galectin-1, pFAK, FAK, pAKT, AKT, in circVPRBP overexpression or control CCa cells, following with or without Dox-induced RACK1 overexpression. **K** Expression of RACK1 was negatively associated with circVPRBP levels in CCa tissue. Two representative cases were shown. Each experiment was performed at least three times independently. **P < 0.01; ***P < 0.001.

Since we have investigated the suppressive role of circVPRBP on tumor growth before, we pursued an in vivo subcutaneous xenograft model to see whether RACK1 was important for the potent anti-proliferative role of circVPRBP in CCa cells. Our results indicated that the overexpression of circVPRBP led to the inhibition in tumor growth, while the Dox-induced RACK1 reversed the inhibition of tumor growth caused by the overexpression of circVPRBP (Fig. 8A). Moreover, the RACK1 could rescue tumor weight and size respectively; these parameters were both suppressed by the overexpression of circVPRBP (Fig. 8B, C). Reconciling with our in vivo assay findings, the Dox-induced RACK1 could abrogate the effects of circVPRBP on the suppression of cell proliferation ability in vitro, as determined by colony formation and EdU assays (Fig. 8D–G).

Meanwhile, the overexpression of RACK1 was shown to rescue the inhibition of invasion in SiHa and MS751 cells that overexpressed circVPRBP (Fig. 8H, I). Similarly, overexpression of RACK1 following the overexpression of circVPRBP led to increased levels of Galectin-1, pAKT and pFAK expression in SiHa and MS751 cells (Fig. 8J). Importantly, negative correlations between low circVPRBP expression and high RACK1 expression were consistently found in our collected CCa patients cohort (Fig. 8K). Collectively, our results strongly suggest that circVPRBP inhibits CCa LN metastasis through promoting degradation of RACK1.

## Discussion

Nowadays, a large class of circRNAs have been identified by high-throughput sequencing and bioinformatics analysis. With the increasing awareness of circRNAs in recent years, researchers have realized the potential role of circRNAs in many critical processes of cancer progression [34–36]. However, the functions of circRNAs in CCa remain largely unclear. In our study, we first identified a circRNA, circVPRBP, played an important suppressive role in the progression of CCa. Subsequently, we demonstrated that circVPRBP was downregulated in cervical cancer tissues and cell lines compared with normal cervix tissues and cells. Moreover, our data showed that that circVPRBP downregulation markedly correlated with poor survival, tumor size, LN metastasis and LVSI. Meanwhile, there has a remarkably negative correlation between circVPRBP expression and micro-lymphatic vessels density in primary CCa tissues. Moreover, circVPRBP expression was an independent prognostic factors for OS and DFS of CCa patients in our cohort, highlighting its applicability as a novel promising prognostic biomarker for cervical cancer.

LN metastasis confers a poor prognosis on cervical cancer patients and lacks effective treatment in the clinic. A better understanding of the molecular mechanisms underlying LN metastasis may assist in identifying patients at high risk for survival and providing effective clinical intervention to cervical cancer. However, little attention has been paid to the understanding why cervical cancers are generally more prone to metastasize to lymph node. Therefore, elucidation of the molecular mechanisms underlying lymphatic metastasis may provide therapeutic strategies for cervical cancer patients with LN metastasis. However, the precise mechanism is largely unknown. Herein, we investigated the crucial role of circRNA in repressing nodal metastasis, thus providing new insight into the interaction of circRNA and metastasis. We observed circVPRBP overexpression notably inhibited lymphangiogenesis and LN metastasis in xenograft lymph node metastasis animal model. Conversely, RfxCas13d induced silencing of circVPRBP promoted lymphangiogenesis and the ability of the cervical cancer cells to metastasize to the LNs in vivo. Therefore, we have reason to believe that low circVPRBP expression supplies a favorable condition for cervical cancer associated LN metastasis.

RACK1 is a highly conserved intracellular adaptor protein that serve as binding sites for multiple interaction partners, and it acts as a scaffolding protein, making it a key mediator of various pathways that contribute to almost every aspect of cellular function [37–39]. Previous studies have highlighted its pivotal function in the cancer related cell biology, and its expression is altered during LN metastasis in cervical cancer [11, 38, 40]. Thus, RACK1 is a crucial factor affecting the development of cancer. In this study, through TRAP assay, we found that circVPRBP could bind RACK1 and promote its proteasome mediated degradation in CCa cells. Meanwhile, the Galectin-1, pAKT and pFAK signaling, which could be regulated by RACK1, had a significant down-regulation upon circVPRBP overexpression. Furthermore, rescue experiments supported the fact that circVPRBP abolished the LN metastasis of cervical cancer depending on the RACK1 destabilization. These findings highlighted the suppressive role of the circVPRBP-RACK1 regulatory axis in the progress of lymphatic metastasis in cervical cancer.

Another important finding in the present study was that we firstly verified a circRNA could influence RACK1 O-GlcNAcylation. It has been reported that O-GlcNAcylation of RACK1 at the amino acid serine122 could promote its stability, thus driving the tumorigenesis of hepatocellular carcinoma [22, 41] and cervical cancer [11, 42] progression. Since RACK1 has the potential to be a theraputic target in cervical cancer LN metastasis, it is pivotal to reach a method accurately shielding O-GlcNAcylation site of RACK1 to destroy its stabilization. In this study, we explored the influence of circVPRBP on O-GlcNAcylation of RACK1, and the results supported the fact that circVPRBP could impede RACK1 binding with O-linked N-acetylglucosamine transferase (OGT), thereby followed by a decrease of RACK1 O-GlcNAcylation and RACK1 degradation. Reconciling with our present findings, recent research reported that a novel antiviral lncRNA EDAL could block the T309 O-GlcNAcylation site of EZH2 to promote EZH2 lysosomal degradation [43]. Here, we identified a new concrete regulatory mechanism of decreased RACK1 O-GlcNAcylation by circVPRBP during cervical cancer LN metastasis, suggesting a promising role of circVPRBP on translational applications for the currently limited treatment of cervical cancer nodal metastasis. Of particular note, these findings reveal that the circRNA mediated protein post-translational modification regulation will be a novel breakthrough in exploring the mechanism of LN metastasis.

**Fig. 9:**
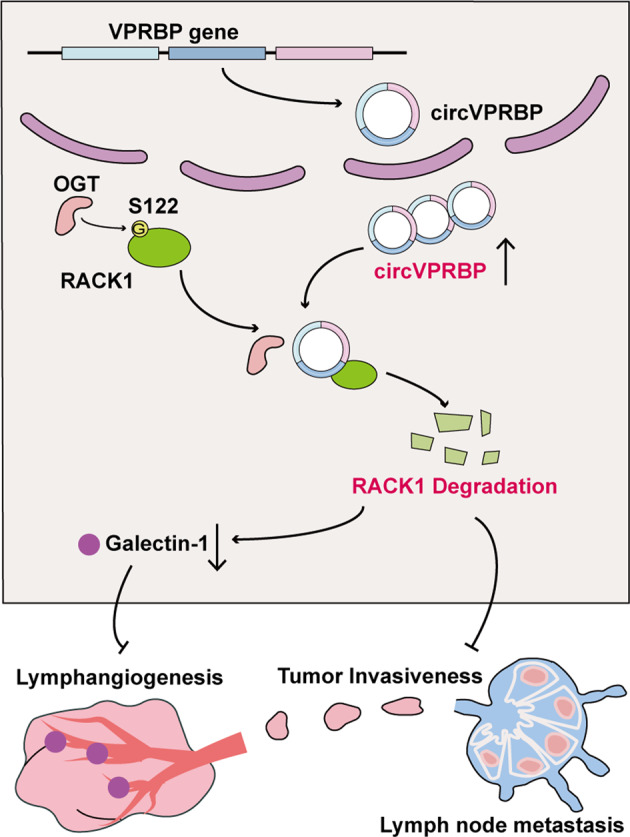
A schematic diagram of the mechanism. circVPRBP could bind to RACK1 and shield the S122 O-GlcNAcylation site to promote RACK1 degradation, resulting in inhibition of Galectin-1 mediated lymphangiogenesis and LN metastasis in CCa.

In conclusion, circVPRBP inhibits lymphangiogenesis and lymph node metastasis through shielding the S122 O-GlcNAcylation site induced RACK1 degradation (Fig. 9). Thus, our findings provide new insights into the mechanism of LN metastasis of cervical cancer and add a promising new target for the development of novel anti-lymphatic metastasis therapeutics.

## Materials and methods

### Cell culture

In this study, we used seven cervical cancer cell lines, including SiHa, HeLa, MS751, C33A, HeLa229, HT-3 and a normal cervical cell H8, all from ATCC and cultured in a humidified atmosphere with 5% CO2 at 37 °C. Human lymphatic endothelial cells (HLECs) were obtained from ScienCell Research Laboratories and cultured in the ECM (ScienCell, CA). All CCa cell lines were cultured in DMEM with 10% fetal bovine serum (Gibco, USA) and 0.5% penicillin/streptomycin (Gibco, China). Cells were cultured in a humid atmosphere with 5% CO_2_ at 37 °C. In 2020, all of the cell lines used were tested for authenticity by short tandem repeat (STR) genotyping; the cell lines were also screened for mycoplasma contamination (e-Myco Mycoplasma PCR Detection Kit; iNtRON).

### Clinical specimens

For qRT-PCR, we collected 30 normal cervical tissues and 50 cervical cancer tissues (containing 25 of LNM negative and 25 of LNM positive); for circRNA in situ hybridization, we obtained 94 cervical cancer (FIGO Stage Ia2, Ib1, Ib2, IIa1 and IIa2) tissues between January 2011 and January 2015 from the First Affiliated Hospital of Sun Yat-sen University (Guangzhou, China). Normal cervical tissues were obtained from the patients who underwent surgery with uterine myoma only. None of the patients were exposed to neoadjuvant therapy before surgery. The specimens were immediately frozen in liquid nitrogen at the time of operation and stored at −80 °C until later use. This study was approved by the Ethical Review Committee of the First Affiliated Hospital of Sun Yat-sen University for the use of these clinical materials for research purposes. All Patients’ samples were obtained according to the Declaration of Helsinki and each patient signed a written informed consent for all the procedures.

### RNA and gDNA extraction, cytoplasmic and nuclear RNA isolation

Total RNAs were extracted from cells or tissues using the SteadyPure Universal RNA Extraction Kit (ACCURATE BIOTECHNOLOGY(Hunan) CO., LTD, Changsha, China) in accordance with the manufacturer’s instructions. gDNA was extracted using the Fastpure Cell/Tissue DNA Isolation Mini Kit (Vazyme, China). Nuclear and cytoplasmic fractions were isolated using a PARIS Kit (Ambion, Life Technologies, USA). RNA extracted from each of the fractions was analyzed by qRT-PCR to determine the levels of nuclear control transcript (U6), cytoplasmic control transcript (GAPDH, Cdr1as), and circVPRBP.

### qRT-PCR, RT-PCR, gel electrophoresis, immunohistochemistry (IHC), western blotting

qRT-PCR, RT-PCR, gel electrophoresis, IHC, western blotting, and HE staining, were performed as described previously [5, 29, 44]. The primary antibodies used in this study are given in Supplementary Table S4. All primers were synthesized by GENEWIZ (Suzhou, China) and primer sequences are given in Supplementary Table S5. Immunostained tissue sections were observed under an optical microscope (Leica, DMI6B, Germany).

### RNase R treatment and Actinomycin D assay

For RNase R treatment experiment, total RNA was extracted and incubated with RNase R (Epicenter Technologies, USA) at 37 °C for 30 min. For actinomycin D assay, cells were incubated with 2 mg/ L actinomycin D (Sigma, USA) for 4, 8, 12 and 24 h. Subsequently, the cells were harvested and extracted RNA at the indicated time. The stability of circVPRBP and its linear transcript VPRBP was measured by qRT-PCR. Every independent experiment was performed in triplicate.

### Plasmid construction and transfection, Lentivirus production and transduction

In order to overexpress circVPRBP ectopically, we cloned the full length of circTADA2A-13 cDNA into the lentiviral pLC5-Puro vector. For generation of RfxCas13d-expressed stable cell lines, p23-NES-RfxCas13d-msfGFP-Flag vector (Addgene #165076) was infected into SiHa and HeLa cells by lentivirus for stable cell line generation. To construct gRNA expression vectors, DNA sequences for gRNAs were synthesized and cloned into pLKO.1-TRC containing direct repeats of each corresponding Cas13. RACK1 S122A mutant plasmid was synthesized by GENEWIZ (Suzhou, China). To generate Dox-inducible RACK1 cell lines, cell lines were infected with lentivirus encoding pLVX-TetOne-RACK1-puro, and then selected with puromycin (2 μg/ ml) for 5 days. Doxycycline hyclate (D9891; Sigma, St. Louis, MO, USA) was dissolved in ddH2O (2 mg/ ml) (in vivo experiment) or added to culture medium at final concentration of 10 µg/ ml in order to induce overexpression of the RACK1. Plasmid transfection was carried out using X-tremeGENE HP DNA Transfection Reagent (Roche, Germany) according to manufacturer’s protocols. For the production of lentiviral particles, lentiX-293T cells were cultured to reach 70% confluence in a 10 cm dish and then cotransfected with an expression vector of interest (10 μg), the psPAX2 vector (7.5 μg) and the pMD2.G vector (2.5 μg). To collect viral particles, the supernatant of cultured lentiX-293T cells was passed through Millipore Millex-GP Filter Unit with 0.45 μm pore size, individually at 48 h and 72 h after transfection. The viral particles were enriched by Lenti-Concentin Virus Precipitation Solution (ExCell Bio) and then resuspended with 1 ml PBS containing 0.1% BSA. Cells were infected with the packaged lentivirus and selected with 2 μg/ ml of puromycin for 5 days.

### Cell proliferation assay

CCK8 and Colony formation assays were performed as previously described [45]. EdU assay was performed by Cell-Light EdU Apollo567 In Vitro Kit (Ribobio, China) according to the manufacture’s protocol.

### Transwell assays and HLECs tube formation assay

For Transwell assays, 50,000 cells were seeded into the upper chamber (Falcon) pre-coated with matrigel (BD, USA) with culture medium but devoid of FBS, while the lower chamber was 500 μl complete medium. After 24–48 h, the cells on the lower surface of the chamber were fixed and then stained. The numbers of migrated cells were counted under microscope. For HLECs tube formation assay, 10,000 HLECs were seeded into 48-well plates (pre-coated with matrigel) containing cell cultre medium and incubated for 10 h. Tube formation was quantified by measuring the total length of tube structures or the number of branch sites/nodes in 3 random fields.

### CircRNA in situ hybridization (ISH)

Formalin-fixed paraffin-embedded tissues were then stained for circVPRBP by in situ hybridization, as previously described [29]. A biotinylated ISH probe was designed by Synbio Tech (Suzhou, China) for hybridization with circVPRBP and signals from the hybridized probes were detected. Staining scores were determined by considering the intensity and proportion of positive cells in five random fields on each tissue section. Scores representing the proportion of positively stained tumor cells in each section were graded as follows: 0, no positive cells; 1, <10% positive cells; 2, 10–50% positive cells; and 3, >50% positive cells. The staining intensity was recorded as 0 (no staining), 1 (weak staining), 2 (moderate staining), and 3 (strong staining). The staining index (SI) was calculated as follows: SI = staining intensity × proportion of positively stained cells; this resulted in scores ranging from 0 to 9. Individual samples were evaluated by two pathologists in a blinded manner, and expression scores of less than or equal to 4 were defined as low expression; samples that were graded as >4 were defined as high expression. For FISH assay, the cy3-labelled circVPRBP probe was designed and synthesized by Geneseed (Guangzhou, China). Sequences of probes were shown in Supplementary Table S5.

### Tagged RNA affinity purification (TRAP) assay and mass spectrometry analysis

TRAP assay was performed as described. CircVPRBP conjugated with MS2 sequence, and MS2 coat protein conjugated with GST plasmids were overexpressed in CCa cells. circVPRBP binding proteins were enriched through GST pulldown assay, and detected by Western blot or mass spectrometry analysis. Eluted proteins were separated by SDS-PAGE and manifested by silver staining. Distinct bands of both circVPRBP-MS2-interacting or MS2-interacting proteins located at the same molecular weight in the gels were separately cut and subjected to mass spectrometry (MS) analysis (Fitgene Biotech, Guangzhou, China). The MS analysis was performed on Q Exactive hybrid quadrupole-Orbitrap mass spectrometer (ThermoFisher Scientific). Protein identification were performed with MASCOT software by searching Uniprot_Aedis Aegypti. MS data was shown in Supplementary Table S6.

### RNA pull down assay

RNA pull-down was performed using a Magnetic RNA-Protein Pull-Down Kit (Cat# 20164, Thermo Fisher Scientific). Different truncated versions of circVPRBP were amplified with a T7 promoter. RNA was transcribed in vitro by TranscriptAid T7 High Yield Transcription Kit (Cat# K0441, Thermo Fisher Scientific) and then labeled with biotin using RNA 3’ End Biotinylation Kit (Cat# 20160, Thermo Fisher Scientific) according to the manufactures’ instructions. 50 pmol of 3’-biotinylated transcribed RNA was incubated with streptavidin magnetic beads and then interacted with cell lysate. The retrieved protein was detected by western blotting. Antisense RNAs of circVPRBP were in vitro transcribed, biotinylated and used as a negative control.

### Co-Immunoprecipitation

Immunoprecipitation (IP) of RACK1 were performed using mouse anti-human RACK1 antibody (1:500, Cat#sc-17754, Santa Cruz) or mouse anti-flag antibody (1:2000, Cat#F3165, Sigma-Aldrich) at 4 °C overnight, followed by incubation with protein A/G magnetic beads (Cat#88802, Thermo Fisher Scientific) and washed with lysis buffer. Mouse IgG (1:100, Cat# 12-371, Millipore) was used as a negative control. The co-immunoprecipitated proteins were heated at 95 °C for 5 min, and then detected by western blotting using rabbit anti-OGT (1:1000, Cat# ab177941, Abcam), rabbit anti-O-linked N-Acetylglucosamine antibody [RL2] (1:1000, Cat# ab2739, Abcam), mouse anti-flag antibody (1:5000, Cat#F3165, Sigma-Aldrich), or rabbit anti-HA (1:1000, Cat# ab18181, Abcam).

### RNA immunoprecipitation (RIP)

RIP assay was performed by Magna RIP^TM^ RNA-binding protein immunoprecipitation kit (Millipore, USA). In brief, magnetic beads were incubated with mouse anti-RACK1 antibody or mouse IgG at room temperature for 30 min to obtain antibody-coated beads. And then nearly 1 × 10^7^ CCa cells were lysed and incubated with antibody-coated on a rotator at 4 °C overnight. The beads were washed, and RNA was extracted from the complexes with RNAiso plus (TaKaRa, Japan) and analyzed by RT-PCR.

### Animal experiments

All animal procedures were approved by the Sun Yat-sen University Animal Care Committee. Female BALB/c nude mice (4–6weeks of age, 18–20 g) were purchased from the Experimental Animal Center of Sun Yat-sen University and raised under SPF conditions. In order to test the effect of circVPRBP on tumor growth in vivo, we injected stable transduced circVPRBP overexpression or control CCa cells into the shoulder of nude mice (1 × 10^7^/100 μl per mouse). Xenograft tumors were monitored every 6–7 days after injection. 30 days later, all mice were sacrificed and all tumors were removed for examining weight. For xenograft mouse lymph node metastatic model, the cells (3 × 10^6^/50 μl per mouse) were inoculated into the foot pad of mice. Primary tumor and lymph nodes were removed, measured and embedded by paraffin for HE or IHC. The tumor and lymph node volumes were calculated using the following formula: Volume(mm^3^) = (length[mm]) × (width[mm])^2^ × 0.52. Simple randomization was used to allocate mice into different groups and no blinding was done.

### Statistical Analysis

SPSS 20.0 statistical software and Prism software (GraphPad Software version 9.3.0) were used for statistical analysis. Unpaired Student *t* test was used to analyze the differences between 2 groups. One-way analysis of variance was applied to evaluate the differences among multiple groups. Overall survival (OS) and disease-free survival curves were calculated with the Kaplan–Meier method and analyzed by log-rank test. Univariate and Multivariate Cox regression analyses were performed to evaluate independent prognostic factors of CCa. The χ2 test and Fisher’s exact test were used to test the relationship between CircVPRBP expression and the clinicopathological parameters. *P*-value < 0.05 was regarded as statistically significant.

## Supplementary information

Supplementary Data

## Data Availability

The datasets generated during and/or analysed during the current study are available from the corresponding author on reasonable request.
