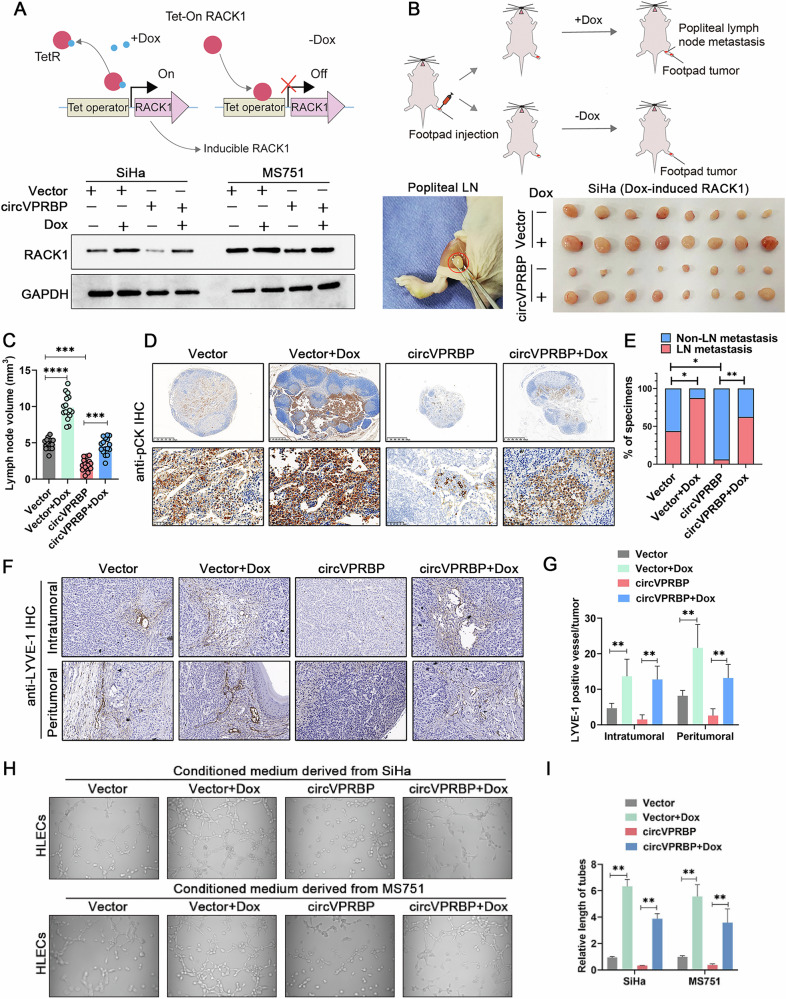# Correction: CircVPRBP inhibits nodal metastasis of cervical cancer by impeding RACK1 O-GlcNAcylation and stability

**DOI:** 10.1038/s41388-025-03440-x

**Published:** 2025-05-15

**Authors:** Chunyu Zhang, Hongye Jiang, Li Yuan, Yuandong Liao, Pan Liu, Qiqiao Du, Chaoyun Pan, Tianyu Liu, Jie Li, Yili Chen, Jiaming Huang, Yanchun Liang, Meng Xia, Manman Xu, Shuhang Qin, Qiaojian Zou, Yunyun Liu, Hua Huang, Yuwen Pan, Jiaying Li, Junxiu Liu, Wei Wang, Shuzhong Yao

**Affiliations:** 1 Department of Obstetrics and Gynecology, the First Affiliated Hospital, Sun Yat-sen University, 510080 Guangzhou, Guangdong China; 2 Department of Biochemistry and Molecular Biology, Zhongshan School of Medicine, Sun Yat-sen University, Guangzhou, 510080 China

**Keywords:** Cervical cancer, Lymphangiogenesis, Metastasis

Correction to: *Oncogene* 10.1038/s41388-023-02595-9, published online 19 January 2023

Following the publication of this article, the authors noted an error in Figure 7H. An incorrect image was inadvertently presented to show HLECs tube formation of circVPRBP-CM group derived from MS751 cells. The correct image has now been included and the corrected figure presented below. The original article has been corrected.

Former Fig 7h:
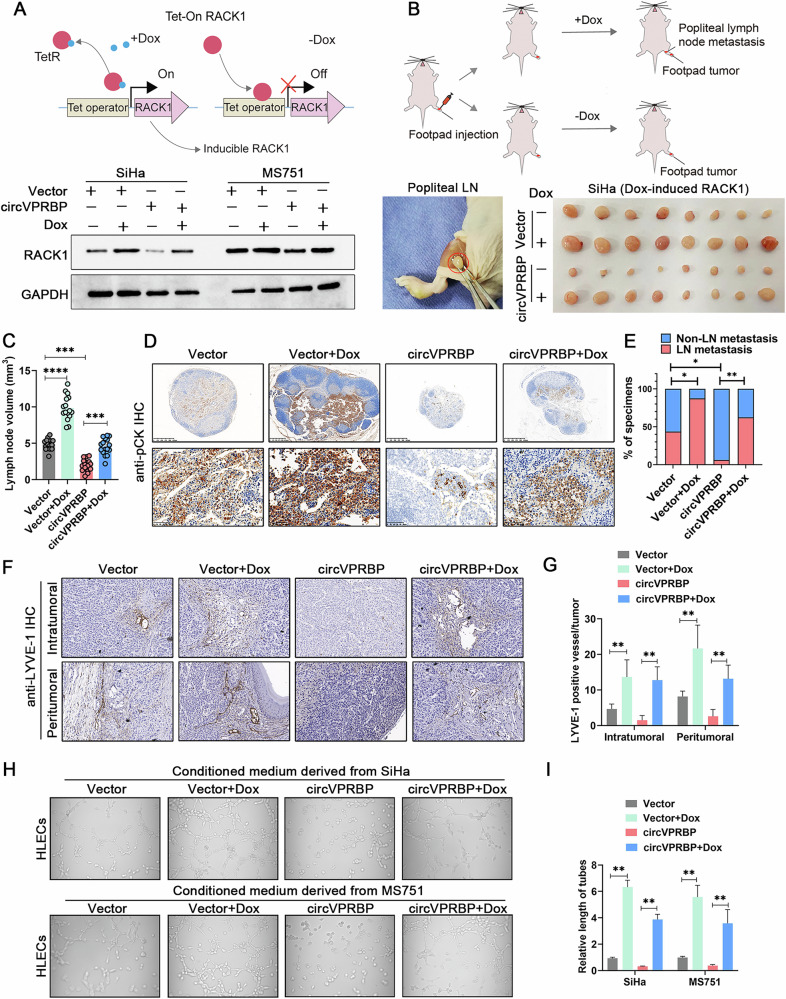


Amended FIg 7h: