# Supplementary material for: CircVPRBP inhibits nodal metastasis of cervical cancer by impeding RACK1 O-GlcNAcylation and stability
Source: Oncogene. 2023 Jan 19;42(11):793–807. doi: 10.1038/s41388-023-02595-9 (PMC10005957; doi:10.1038/s41388-023-02595-9)
Supplement: Supplementary file 1 — Supplementary Data [file 41388_2023_2595_MOESM1_ESM.pdf]

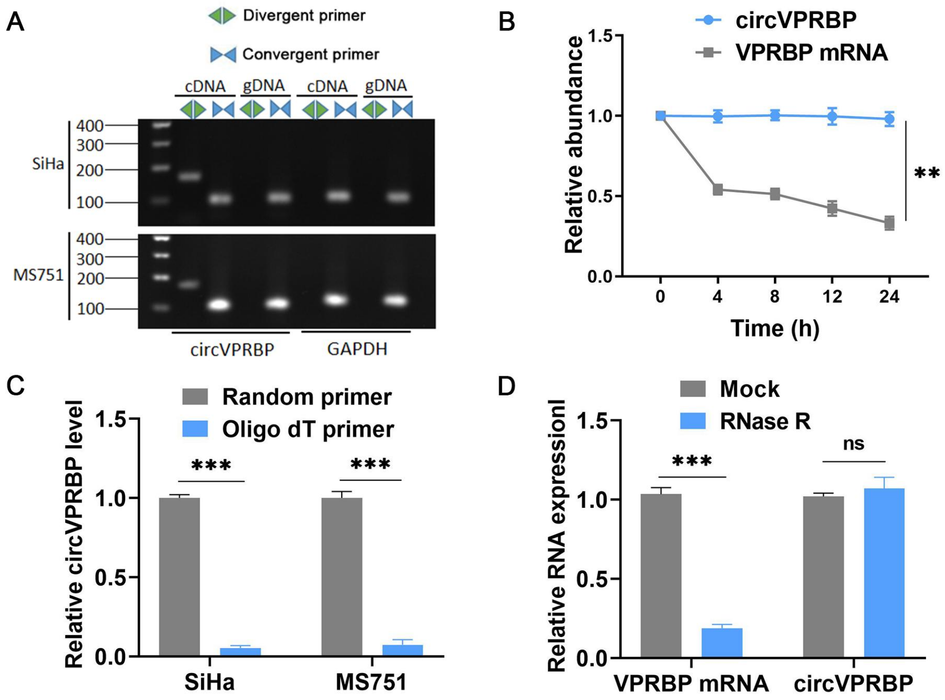

Figure S1. Characterization of circVPRBP in cervical cancer cells

A PCR analysis for circVPRBP and its linear counterpart in cDNA and genomic DNA in SiHa and MS751 cell lines. **B** RT-qPCR analysis for the abundance of circVPRBP and VPRBP mRNA treated with Actinomycin D at the indicated points. **C** Random and oligo dT primers were used to analyzed the abundance of circVPRBP in reverse transcription experiments. **D** RT-qPCR analysis for the expression levels of circVPRBP and VPRBP mRNA treated with RNase R. Each experiment was performed at least three times independently. ns, no significant; $**P < 0.01$; $***P < 0.001$.

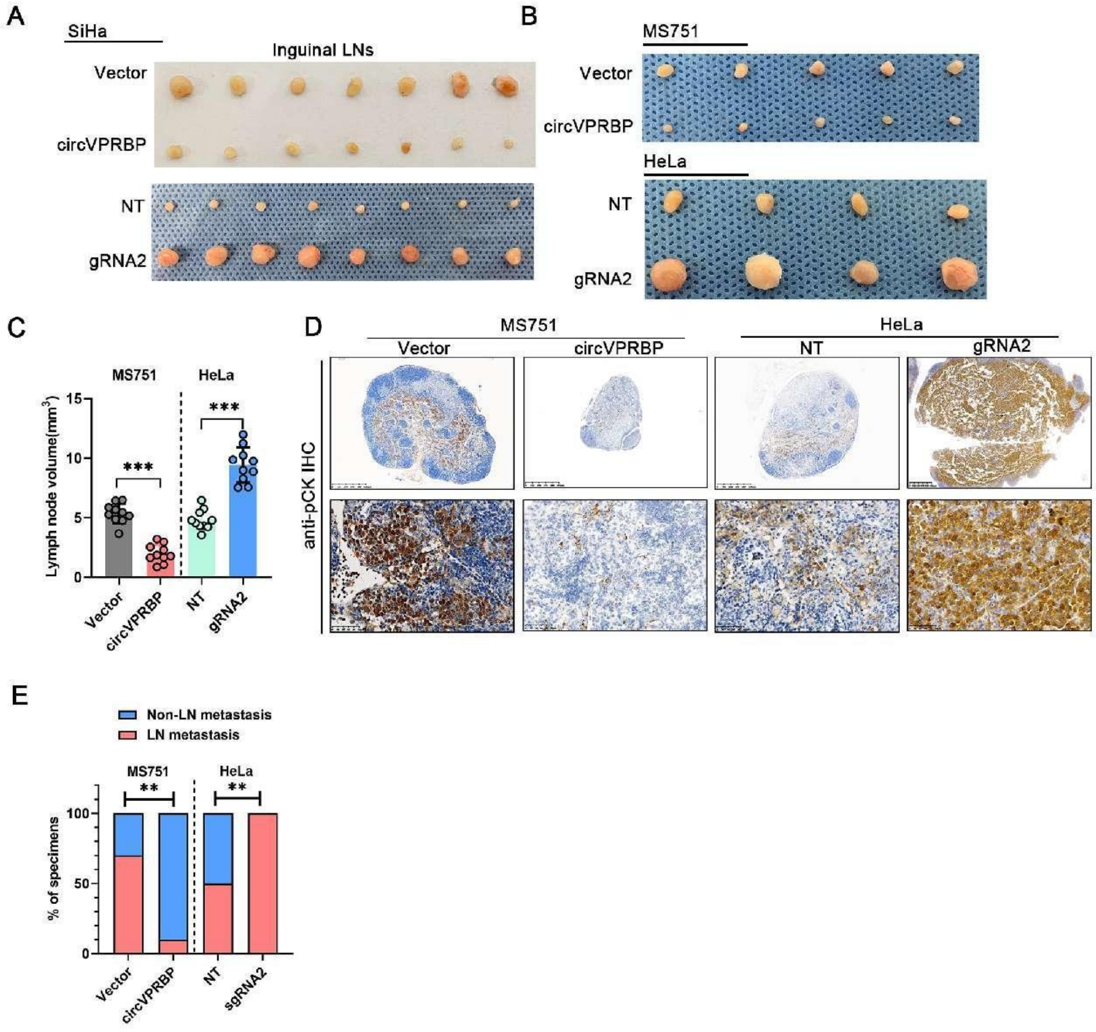

Figure S2. circVPRBP inbihits CCa LN metastasis in vivo

A and **B** Representative images of metastatic LNs in indicated groups. **C** Quantification of the lymph node volume in different groups. **D** Representative images of immunostaining of pan-cytokeratin of popliteal LNs. **E** Quantification of the rate of LN metastasis in the indicated groups. **P < 0.01; ***P < 0.001.

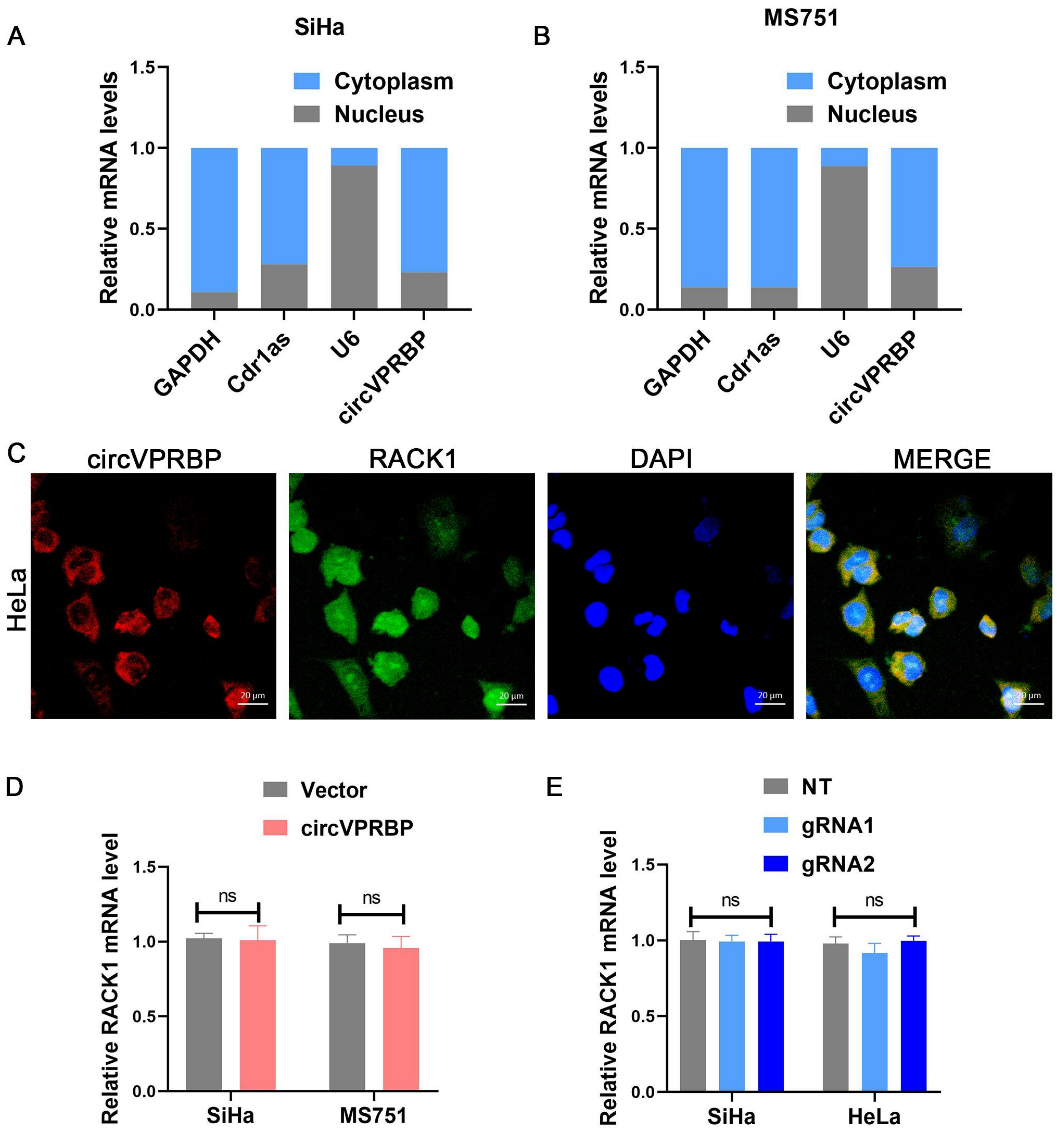

Figure S3. A, **B** Cytoplasmic and nuclear mRNA fractionation experiments in SiHa and MS751 cells showing that circVPRBP was localized mainly in the cytoplasm. GAPDH, Cdr1as, and U6 were applied as positive controls in the cytoplasm and nucleus, respectively. **C** Fluorescence colocalization assay showed that circVPRBP and RACK1 protein colocalize in the cytoplasm of HeLa cells. **D, E** mRNA levels of RACK1 in indicated CCa cells. ns, no significant.

Table S1. Correlation between circVPRBP expression with clinicopathologic characteristics of patients in cervical cancer

Characteristics	Total	circVPRBP expression		*P*-value
	94	Low	High	
Age (years)				0.104
< 42	40	20	20	
≥ 42	54	36	18	
FIGO stage				0.583
I (Ia2+Ib1+Ib2)	72	44	28	
II (IIa1+IIa2)	22	12	10	
Tumor size (cm)				**0.023**
≤ 4	73	39	34	
> 4	21	17	4	
Pathologic types				0.291
Squamous cell carcinoma	73	45	28	
Adenocarcinoma	16	7	9	
Adenosquamous carcinoma	5	4	1	
Differentiation				0.357
Well	6	3	3	
Moderate	40	21	19	
Poor	48	32	16	
Stromal invasion				0.270
< 1/2	58	32	26	
≥ 1/2	36	24	12	
LVSI				**0.020**
Positive	15	13	2	
Negative	79	43	36	
LNM				**0.014**
Positive	19	16	3	
Negative	75	40	35	
Vaginal invasion				0.270
Positive	3	3	0	
Negative	91	53	38	
Parametrial invasion				0.513
Positive	2	2	0	
Negative	92	54	38	

FIGO: the International Federation of Gynecology and Obstetrics; LVSI, lymphovascular space invasion; LNM, lymph node metastasis.

Table S2. Univariate Cox's proportional hazards model analysis of disease-free survival and overall survival

Variables	Total	OS	DFS
	94	*P*-value	*P*-value
Age (years)		0.186	0.255
< 42	40		
≥ 42	54		
FIGO stage		0.917	0.882
I (Ia2+Ib1+Ib2)	72		
II (IIa1+IIa2)	22		
Tumor size (cm)		**0.003**	**0.005**
≤ 4	73		
> 4	21		
Pathologic types		0.781	0.640
Squamous cell carcinoma	73		
Adenocarcinoma	16		
Adenosquamous carcinoma	5		
Differentiation		0.657	0.351
Well	6		
Moderate	40		
Poor	48		
Stromal invasion		0.034	0.008
< 1/2	58		
≥ 1/2	36		
LVSI		**0.021**	**0.017**
Positive	15		
Negative	79		
LNM		**0.004**	**0.002**
Positive	19		
Negative	75		
Vaginal invasion		0.418	0.524
Positive	3		
Negative	91		
Parametrial invasion		0.511	0.462
Positive	2		
Negative	92		
circVPRBP		**0.014**	**0.010**
Low	56		
High	38		

FIGO: the International Federation of Gynecology and Obstetrics; LVSI, lymphovascular space invasion; LNM, lymph node metastasis.

Table S3. Multivariate Cox's proportional hazards model analysis of disease-free survival and overall survival in cervical cancer patients

Variables	Overall survival		Disease-free survival	
	HR (95% CI)	*P*	HR (95% CI)	*P*
Tumor size (cm)		**0.002**		**0.002**
≤ 4 (reference)	1		1	
> 4	4.888 (1.798-13.293)		4.345 (1.752-10.777)	
LVSI		**0.015**		**0.004**
Negative (reference)	1		1	
Positive	3.456 (1.271-9.398)		3.927 (1.559-9.890)	
LNM		**0.011**		**0.010**
Negative (reference)	1		1	
Positive	3.510 (1.327-9.285)		3.101 (1.305-7.369)	
circVPRBP		**0.027**		**0.024**
Low (reference)	1		1	
High	0.181 (0.040-0.825)		0.238 (0.068-0.829)	

HR, hazard ratio; 95% CI, 95% confidence interval; LNM, lymph node metastasis; LVSI, lymphovascular space invasion; For the stepwise multivariate analysis, forward LR method was used to select significant variables. Variables entered for multivariate analysis were the following: tumor size, Stromal invasion, LNM, LVSI, circVPRBP.

Table S4. Antibodies used in the study

Target protein	Antibody	Application in analysis
AKT	CST, #4691S	WB (1:1000)
p-AKT	CST, #4060S	WB (1:1000)
FAK	Proteintech, #12636-1-AP	WB (1:1000)
pFAK	CST, #3283S	WB (1:1000)
Galectin-1	Abcam, #ab108389	WB (1:10000)
RL2	Abcam, #ab2739	WB (1:1000)
OGT	Abcam, #ab177941	WB (1:1000)
GST	Proteintech, #66001-2-Ig	WB (1:6000)
HA	Abcam, #ab18181	WB (1:1000)
Flag	Sigma-Aldrich,#F3165	WB(1:5000)；IP（1:2000）
GAPDH	Proteintech, #10494-1-AP	WB (1:10000)
HRP-conjugated Affinipure Goat Anti-Rabbit IgG (H+L)	Proteintech, #SA00001-2	WB (1:10000)
HRP-conjugated Affinipure Goat Anti-Mouse IgG (H+L)	Proteintech, #SA00001-1	WB (1:10000)
Pan-cytokeratin	CST, #4545S	IHC (1:400)
LYVE-1	Abcam, #ab14917	IHC (1:200)
RACK1	SantaCruz, #sc-17754	WB(1:500); IP (2 μg per 200 μg of total protein)
RACK1	Proteintech, #27592-1-AP	IHC and IF (1:800)

Table S5. Sequences of primers, gRNAs and probes used in the study

**Primer for PCR, RT-qPCR and RIP**		
CircVPRBP (divergent)	**F**	5'-GGAGACATTGCCCACATTCC-3'
	**R**	5'-CTCCTCCTGTCCACTAAACAC-3'
CircVPRBP (convergent)	**F**	5'-GCGGATGCTTTGATAGGCACCT-3'
	**R**	5'-ACAGCAGGTGAAGCCACTCTCA-3'
RACK1	**F**	5'-ACCATCATCATGTGGAAACTGAC-3'
	**R**	5'-GTGCCCGTTGTGAGATCCC-3'
Galectin-1	**F**	5'-TCGCCAGCAACCTGAATCTC-3'
	**R**	5'-GCACGAAGCTCTTAGCGTCA-3'
GAPDH	**F**	5'-AGAAGGCTGGGGCTCATTTG-3'
	**R**	5'-AGGGGCCATCCACAGTCTTC-3'
CDR1as	**F**	5'-ACGTCTCCAGTGTGCTGA-3'
	**R**	5'-CTTGACACAGGTGCCATC-3'
GAPDH (divergent)	**F**	5'-TGTACCATCAATAAAGTACCCTGTG-3'
	**R**	5'-AAATCCGTTGACTCCGACCT-3'
U6	**F**	5'-ACAGATCTGTCGGTGTGGCAC-3'
	**R**	5'-GGCCCCGGATTATCCGACATTC-3'
**gRNAs for cirVPRBP and ISH probe**		
NT		5'-GCAGGGTTTTCCCAGTCACGACGTTGTAAA-3
gRNA1		5'-AAATAGGACGGAATGTGGGCAAT-3
gRNA2		5'-GAAATAGGACGGAATGTGGGCAA-3
gRNA3		5'-AATAGGACGGAATGTGGGCAATG-3
Digoxigenin-labeled circVPRBP probe for ISH		5'-AAATAGGACGGAATGTGGGCAATGTCTCCTTTTG-3'
Cy3-labeled circVPRBP probe for FISH		5'-ATAGGACGGAATGTGGGCAATGT- 3'
**Primers for in *vitro* transcription**		
circVPRBP(1-367)	**F**	5'-TAATACGACTCACTATAGGGATTCCGTCCTATTTCAGTG-3'
	**R**	5'-GTGGGCAATGTCTCCTTTTGTGC-3'
circVPRBP(1-122)	**F**	5'-TAATACGACTCACTATAGGGATTCCGTCCTATTTCAGTG-3'
	**R**	5'-AGAGCTTCAGCTGCCCTGTGC-3'
circVPRBP(122-182)	**F**	5'-TAATACGACTCACTATAGGGTATAATGTGTTTAGTGGACAGG-3'
	**R**	5'-GTGTGATGGCTGAGTTGTG-3'
circVPRBP(1-182)	**F**	5'-TAATACGACTCACTATAGGGATTCCGTCCTATTTCAGTG-3'
	**R**	5'-GTGTGATGGCTGAGTTGTG-3'
circVPRBP(182-367)	**F**	5'-TAATACGACTCACTATAGGGCATCTTGAACCTTCCAGGG-3'
	**R**	5'-GTGGGCAATGTCTCCTTTTGTGC-3'
circVPRBP(antisense)	**F**	5'-TAATACGACTCACTATAGGGGTGGGCAATGTCTCCTTTTGTGC-3'
	**R**	5'-ATTCCGTCCTATTTCAGTGTTCC-3'

Table S6. circVPRBP-interacting proteins identified by mass spectrometry analysis.

number	protein	mass
1	sp|Q96E11|RRFM_HUMAN	29373
2	sp|P61604|CH10_HUMAN	10925
3	sp|P06702|S10A9_HUMAN	13291
4	sp|Q9UI30|TR112_HUMAN	14304
5	sp|P62318|SMD3_HUMAN	14021
6	sp|Q04837|SSBP_HUMAN	17249
7	sp|Q8NAV1|PR38A_HUMAN	37681
8	sp|P41208|CETN2_HUMAN	19726
9	sp|Q14320|FA50A_HUMAN	40216
10	sp|P49411|EFTU_HUMAN	49852
11	sp|P63244|RACK1_HUMAN	35511
12	sp|P82979|SARNP_HUMAN	23713
13	sp|O95843|GUC1C_HUMAN	23864
14	sp|P06576|ATPB_HUMAN	56525
15	sp|Q15056|IF4H_HUMAN	27425
16	sp|P13984|T2FB_HUMAN	28420
17	sp|Q13112|CAF1B_HUMAN	61910
18	sp|Q8WXX5|DNJC9_HUMAN	30062
19	sp|P35232|PHB_HUMAN	29843
20	sp|Q7Z7K6|CENPV_HUMAN	30383
21	sp|P52907|CAZA1_HUMAN	33073
22	sp|Q8IVM0|CCD50_HUMAN	35914
23	sp|O95400|CD2B2_HUMAN	37737
24	sp|Q9H6T3|RPAP3_HUMAN	75957
25	sp|P12429|ANXA3_HUMAN	36524
26	sp|Q9UBP9|GULP1_HUMAN	34925
27	sp|Q96EI5|TCAL4_HUMAN	24746
28	sp|Q8IZL9|CDK20_HUMAN	38898
29	sp|Q9NVD7|PARVA_HUMAN	42274
30	sp|P12277|KCRB_HUMAN	42902
31	sp|Q6IN84|MRM1_HUMAN	39070
32	sp|Q99706|KI2L4_HUMAN	41974
33	sp|Q9BXN1|ASPN_HUMAN	43788
34	sp|Q15084|PDIA6_HUMAN	48490
35	sp|Q7L4I2|RSRC2_HUMAN	50586
36	sp|Q2TAY7|SMU1_HUMAN	58134
37	sp|Q02413|DSG1_HUMAN	114702
38	sp|P12268|IMDH2_HUMAN	56226
39	sp|Q9UBS0|KS6B2_HUMAN	53821
40	sp|Q16630|CPSF6_HUMAN	59344
41	sp|Q8N884|CGAS_HUMAN	59462
42	sp|P36915|GNL1_HUMAN	69132
43	sp|Q07065|CKAP4_HUMAN	66097
44	sp|P49368|TCPG_HUMAN	61066
45	sp|P04040|CATA_HUMAN	59947
46	sp|Q9H6Z4|RANB3_HUMAN	60515
47	sp|O15460|P4HA2_HUMAN	61263
48	sp|O60684|IMA7_HUMAN	60733
49	sp|Q96KC8|DNJC1_HUMAN	64185
50	sp|P61764|STXB1_HUMAN	67925
51	sp|P19338|NUCL_HUMAN	76625
52	sp|Q8IXB1|DJC10_HUMAN	92333
53	sp|Q08J23|NSUN2_HUMAN	87214
54	sp|P14923|PLAK_HUMAN	82434
55	sp|Q3YEC7|RABL6_HUMAN	79785
56	sp|Q9Y4R7|TTLL3_HUMAN	88612
57	sp|Q08554|DSC1_HUMAN	101406
58	sp|Q9H6N6|MYH16_HUMAN	128439
59	sp|Q14566|MCM6_HUMAN	93801
60	sp|O75150|BRE1B_HUMAN	114350
61	sp|Q5BJF6|ODFP2_HUMAN	96140
62	sp|Q9UKJ3|GPTC8_HUMAN	165010
63	sp|Q96HA7|TONSL_HUMAN	153001
64	sp|Q9BX26|SYCP2_HUMAN	177239
65	sp|P51805|PLXA3_HUMAN	211049
66	sp|P35579|MYH9_HUMAN	227646
67	sp|Q07283|TRHY_HUMAN	254233
68	sp|Q5H9R4|ARMX4_HUMAN	237416
